# Supplementary material for: Context-Dependent Effects Explain Divergent Prognostic Roles of Tregs in Cancer
Source: Cancers (Basel). 2022 Jun 17;14(12):2991. doi: 10.3390/cancers14122991 (PMC9221270; doi:10.3390/cancers14122991)
Supplement: Supplementary file 1 [file cancers-14-02991-s001.zip › cancers-1744153-supplementary.pdf]

cancers

MDPI

Supplementary Materials: Context-dependent effects explain-divergent prognostic roles of Tregs in cancer

Elise Amblard and Vassili Soumelis

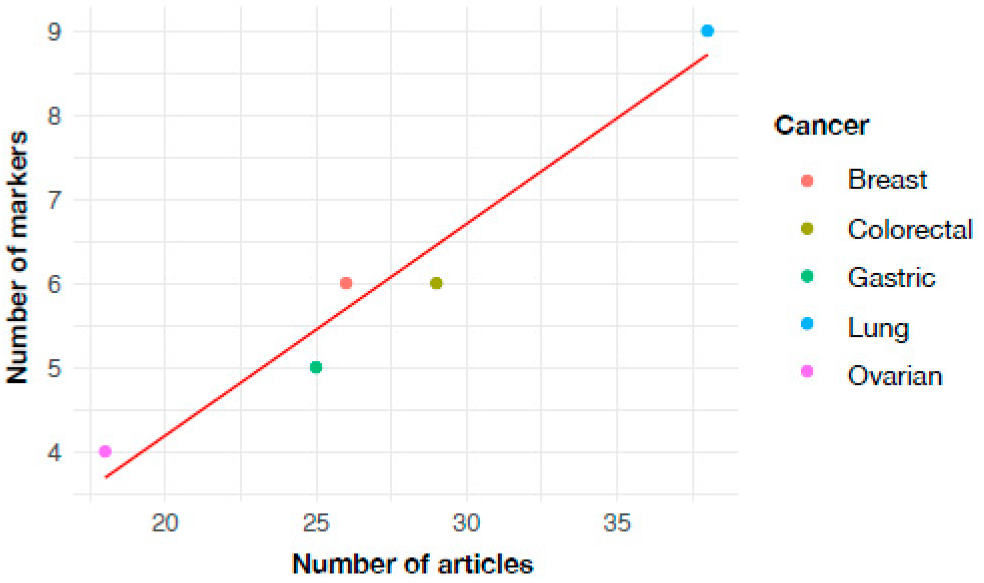

Figure S1. Correlation between the number of markers used and the number of articles, by cancer type.

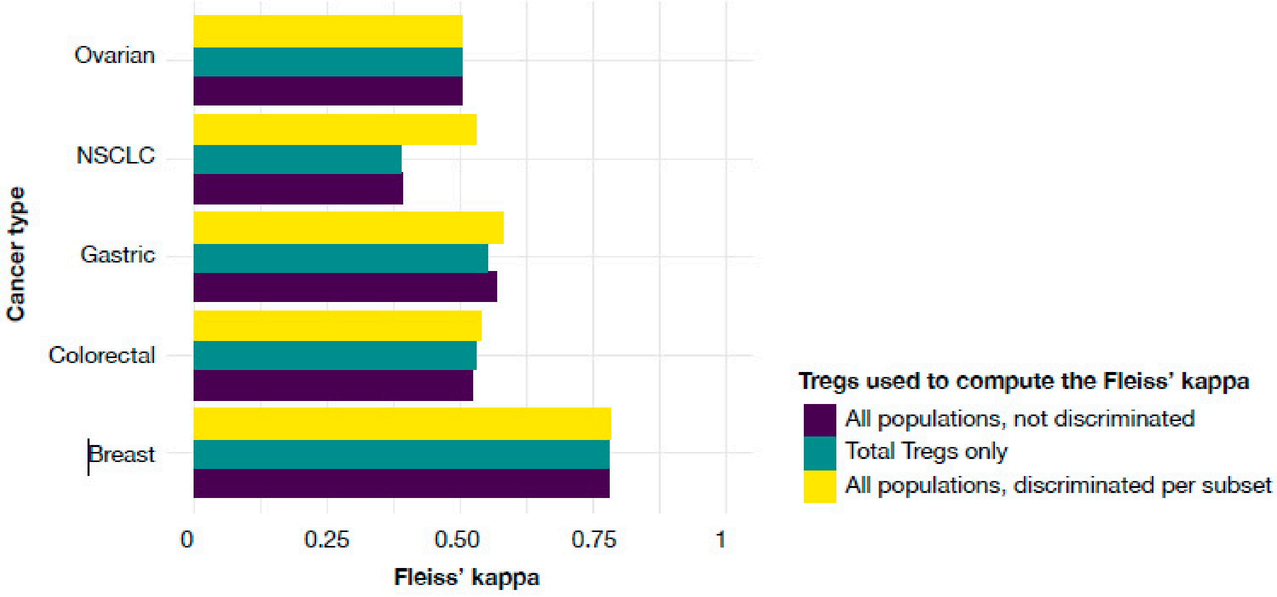

Figure S2. Fleiss' kappa to determine the degree of agreement between articles, stratified by regulatory subset.

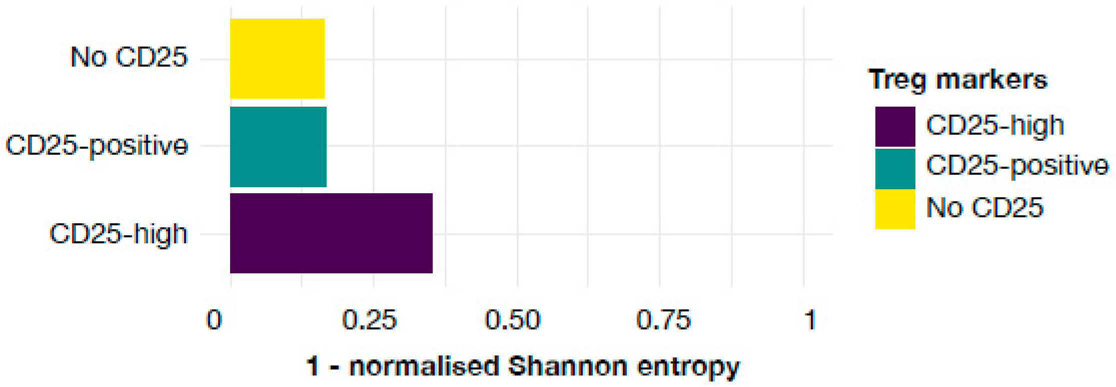

Figure S3. Shannon entropy for articles defining Tregs based on expression of the CD25 marker.

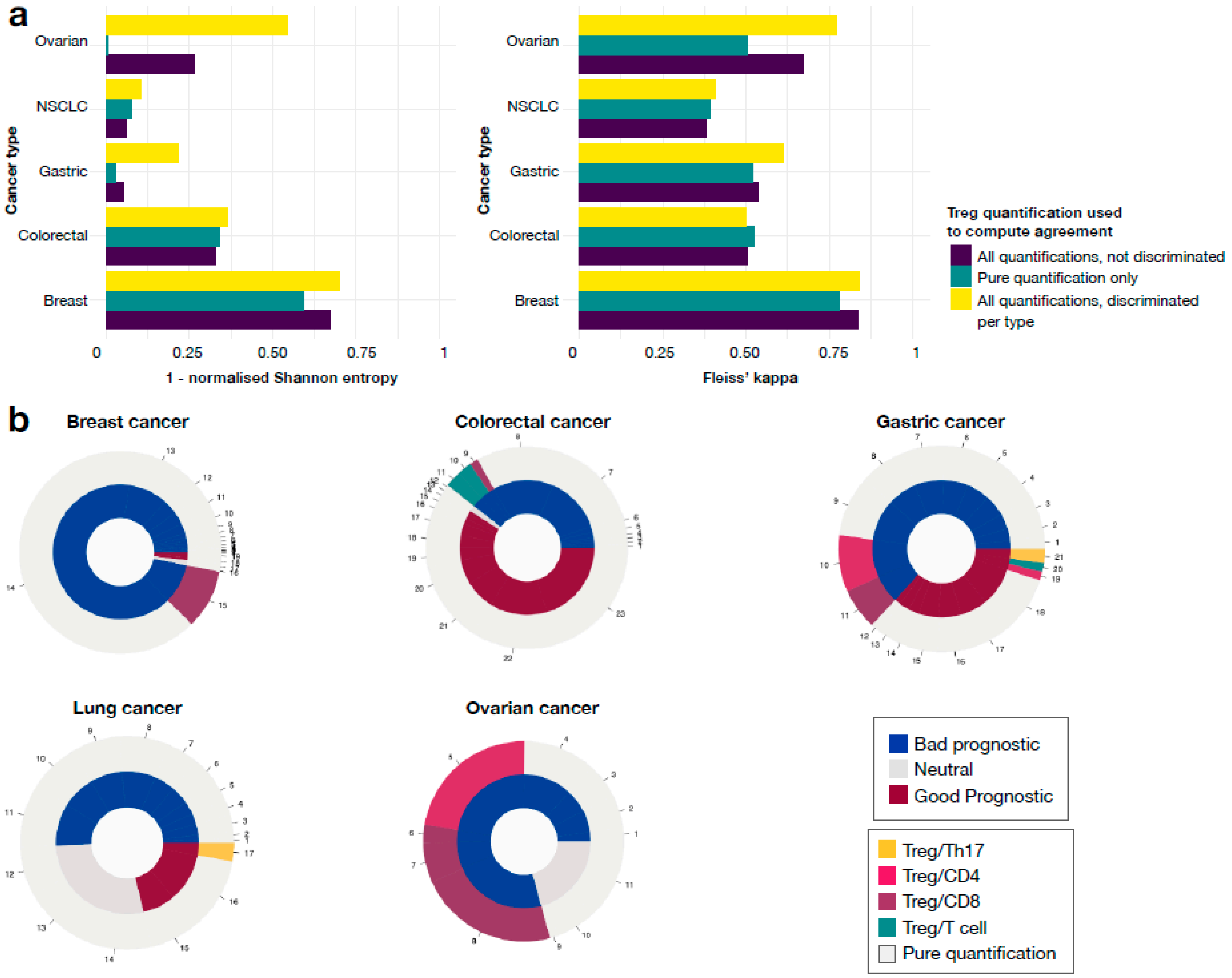

Figure S4. Impact of the mode of Tregs quantification on the prognostic value. (**a**) Agreement between articles, stratified by type of quantification: Shannon entropy (left), Fleiss' kappa (right). (**b**) Pie chart of prognosis with information about quantification, by cancer type. Each portion is an article and its size reflects the number of patients included.

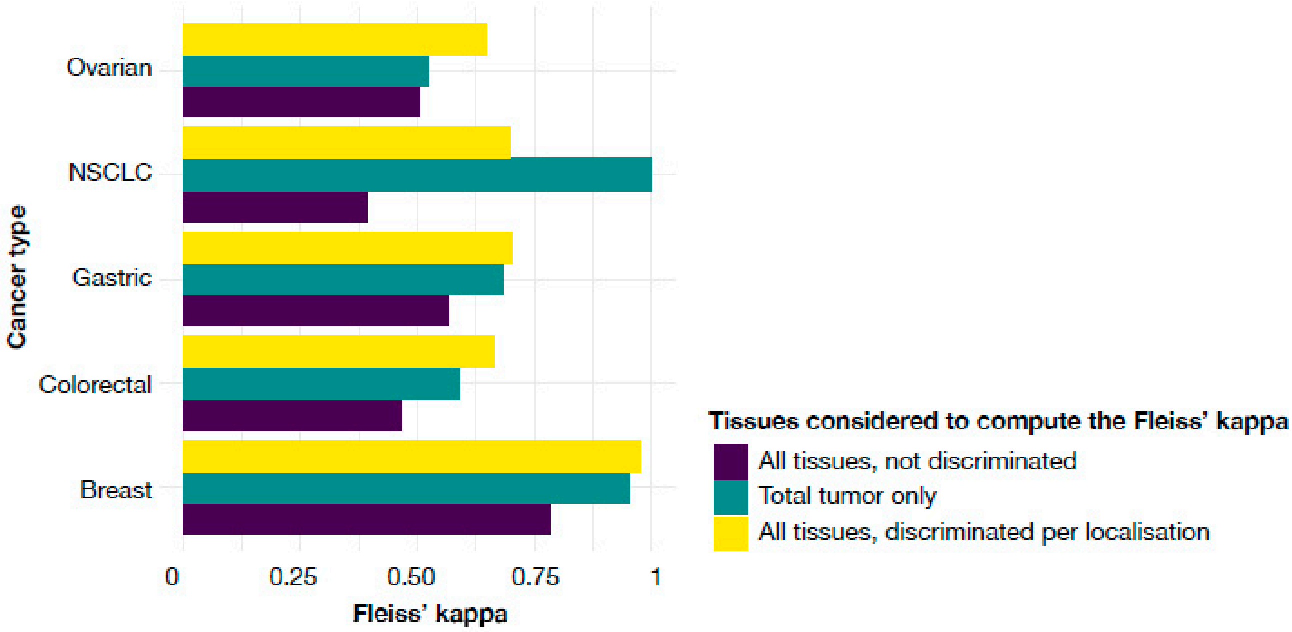

Figure S5. Fleiss' kappa for assessing the degree of agreement between articles, stratified by anatomic location.

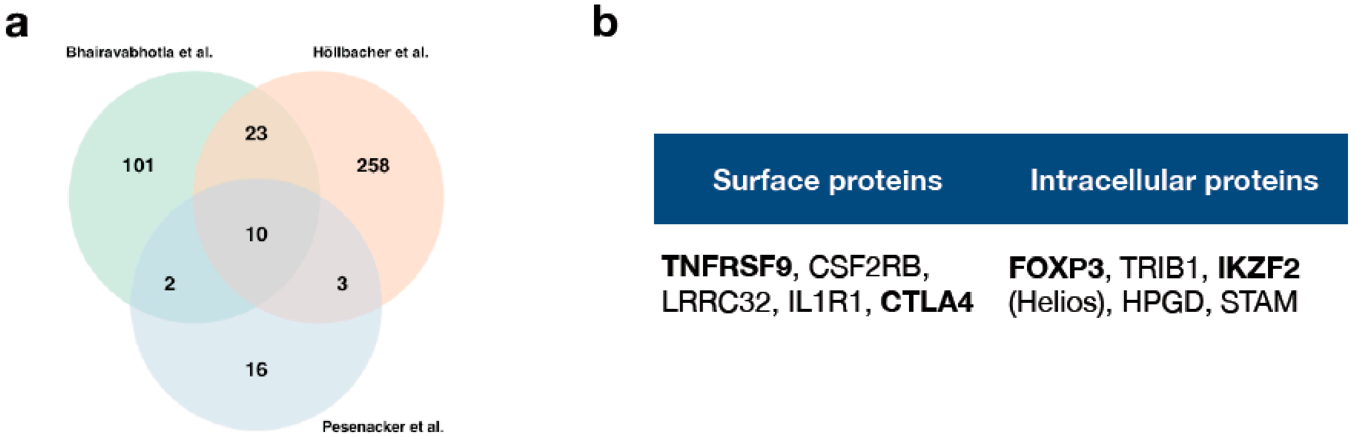

Figure S6. Overlap between transcriptomic regulatory signatures. (**a**) Venn diagram of the gene signature forTregs from Bhairavabhotla et al. [2016], Hollbacher et al. [2020], Pesenacker et al. [2016]. (**b**) Genes at the intersection of the three signatures. Genes in bold have been repeatedly linked to the regulatory phenotype.

Table S1. List of articles used in this analysis with PMID references.

Cancer	Good prognosis	Neutral prognosis	Poor prognosis	Undetermined
Breast	28233108, 23075422	22842982, 25849846	24124553, 18820666, 17135638, 27851913, 23529839, 20181533, 23836289, 18413832, 23026134, 21717105, 22842982, 22842982, 27566250, 24562936	28388539, 17135638, 22760213, 27851913, 23529839, 20181533, 22836755, 19855964, 22116346,23712790, 18294387
Colorectal	20386463, 19064967, 24005418, 19856313,	23382847, 16740757	25268580, 26298011, 19064967, 21915633,	17205133, 22276195, 22319577, 23382847,

	24675384, 24997850		24005418, 22907255, 19577568, 19908042, 31681276	23613769, 25268580, 24064667, 20952660, 24005418, 22907255, 27851914, 22207629, 18985040, 25405854
Gastric	23807713, 24331841, 24170095, 21347781, 19732435, 32204925, 266799288		29804142, 28817117, 27756099, 24657498, 24040244, 22374482, 22083420, 21792941, 20221835, 19900843, 19153062	29804142, 28817117, 26782287, 24261990, 24170095, 22083420, 21528082, 21347781, 20422211, 19900843, 19153062, 18224687, 18087278
Lung	22300751, 23335103, 279767333	22300751, 23305175, 23891508	17099880, 20234320, 21719142, 22363469, 22608141, 23305175, 23891508, 27773662, 27851914,279767331, 279767332	15846066, 16698419 17163448, 17825949, 18771959, 19148592, 19332094, 19597336, 21258248, 21611754, 21663645, 22363469, 24345703, 24780112, 26042578, 26280204, 26541534, 27000869, 27474372, 27866241, 28513867, 28731226
Ovarian		26077607, 20006900, 18314181	27748885, 26298430, 25365237, 24244610, 17875732, 16344461, 32902402	28437737, 27759594, 26482613, 25514665, 25416072, 23948613, 22865582, 22798340, 18166500, 18036640
